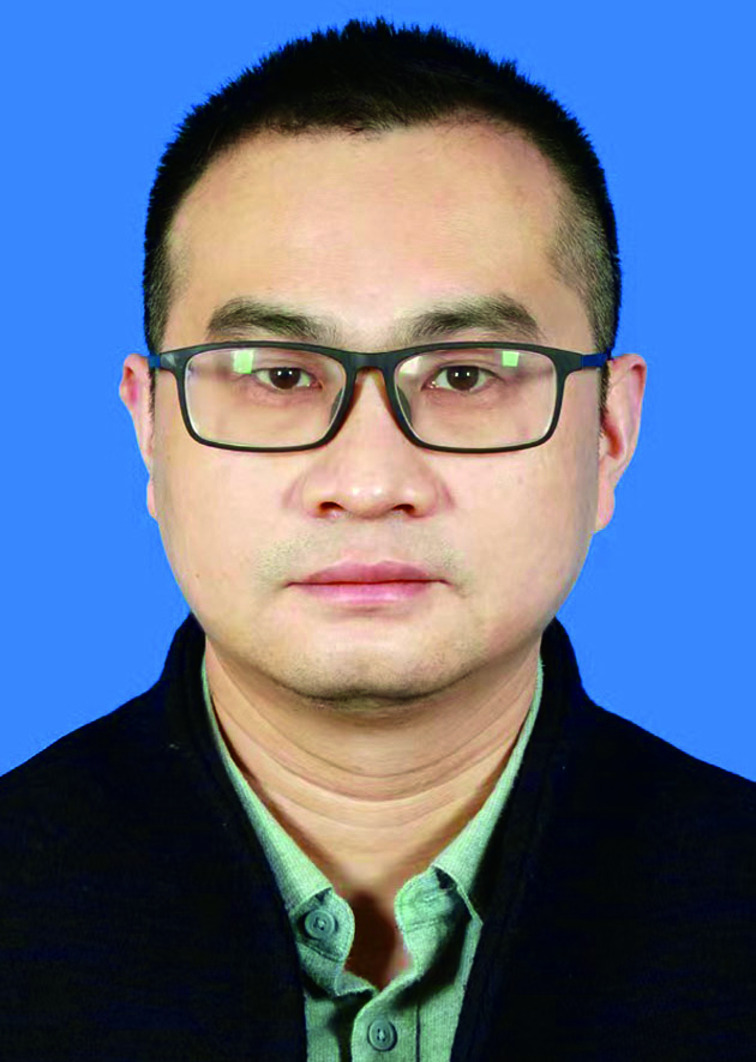# Artificial Spidroin Nanogenerator‐Based Articulus Wound Dressing

**DOI:** 10.1002/open.202400257

**Published:** 2024-10-30

**Authors:** Xiaoming Ma, Shuhuan Li, Bingbing Gao

**Affiliations:** ^1^ Department of Orthopedics Taizhou People's Hospital Taizhou, Jiangsu Province People's Republic of China; ^2^ School of Pharmaceutical Sciences College of Biotechnology and Pharmaceutical Engineering Nanjing Tech University Nanjing 211816 China

**Keywords:** Artificial Spidroin, Nanogenerator, Wound Dressing

## Abstract

Articulus wound infection is a threat to human health. Existing medical materials have poor biocompatibility and may contain harmful chemicals, causing allergies and secondary infections. Therefore, there is an urgent need to develop innovative medical materials. Materials made of artificial spider silk proteins have been widely used in wound healing because of their good biocompatibility, biodegradability, cell adhesion and bioelectronic properties.

## Graphical abstract

It is urgent to develop innovative medical materials for articulus wound healing. Artificial spider silk proteins have been widely used in articulus wound healing for good biocompatibility, biodegradability, cell adhesion and bioelectronic properties.

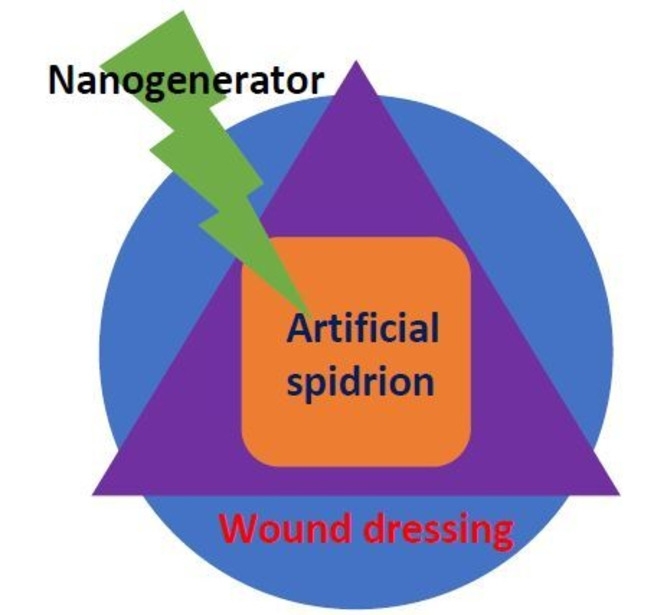

The skin is the organ with the largest surface area of the human body. The damage or loss of skin tissue caused by trauma is called a wound, and a wound that cannot be repaired normally and completely for a long period of time is defined as a chronic wound.^[1]^ Chronic wounds are affected by hyperglycemia, related growth factor deficiency, peripheral vascular diseases and other pathological factors, and the healing of chronic wounds is more difficult than that of ordinary wounds.^[2]^ Nonhealing wounds can cause pain, inflammation, bacterial infection and even death, posing a major threat to human health. Therefore, to treat chronic wounds, new multifunctional dressings are being developed. Traditional dressings mainly use polymer compounds that cover wounds, absorb exudates and provide protection but lack biodegradability^[3]^ and may release microplastics,[^4^, ^5^] posing a threat to humans and the environment.^[6]^ In addition, allergic reactions, skin damage[^7^, ^8^] and secondary infections^[9]^ may occur during application, possibly related to harmful chemicals in the dressing.^[10]^ Compared with silk protein, spider silk protein has the advantage that its heterologous expression has been studied extensively, and its production in *Escherichia coli* has made great progress.^[11]^ In addition, research on the sequence design and modification of spider silk proteins is relatively mature and has advantages in the development of multifunctional spider silk proteins.^[12]^ Moreover, because spider silk protein is obtained through heterogenic expression, it does not need to dissolve similarly to silk, and because its protein exists directly in liquid form, it has good plasticity and can be used in the preparation of medical materials with different needs.^[13]^

In recent years, wound dressings prepared from artificial spidroins have attracted extensive attention because of their unique advantages, such as good biocompatibility, biodegradability, good mechanical properties^[14]^ and excellent bioelectronic performance.^[12]^ With the increasing demands for personalized medicine, the demand for smart wound dressings is also increasing, and spidroin smart wound dressings have great potential in the next generation of wound management. Owing to their excellent bioelectronic performance, in the process of wound treatment, spidroin bioelectronic dressings can generate appropriate current stimulation to promote wound repair and provide a new approach for the preparation of battery‐free intelligent wound dressings. In addition, excellent mechanical properties enable spidroin bioelectronic dressings to adapt to the complexity and challenges of the active site wound environment.^[13]^ Moreover, owing to the good biodegradability of spidroin bioelectronic dressings, they can effectively reduce the impact of environmental problems on human health. In conclusion, the use of smart wound dressings prepared from spidroins is a promising approach for smart wound management.

Spider silk is a type of high‐molecular‐weight protein fiber. Its exceptional mechanical properties and biocompatibility herald a vast and promising application in the future. Currently, the genetic codes for many spidroins have been elucidated. The sequence design, expression strategies, and application development of artificial spidroins have garnered extensive research interest;[^14^, ^15^, ^16^] however, as spiders are cannibalistic arthropods, they cannot be bred on a large scale such as silkworms, making heterologous expression the sole means to obtain significant quantities of spider silk.^[17]^ Researchers have conducted comprehensive exploratory studies on dissecting and modifying the sequence motifs and structural characteristics of spidroins. In general, spider silk proteins are composed of three parts: the more conserved N‐terminal (NT) nonrepeat region, the C‐terminal (CT) nonrepeat region, and the highly specific repeat core region. Recent studies have shown that N/CT is related to the self‐assembly of spider silk proteins and plays a leading role in conformational transformation during the spinning process.[^18^, ^19^] The repeat core region is directly related to the spinning performance of spider silk proteins, and the repeat core regions of different types of glandular filaments greatly differ, resulting in significant differences in spinning performance.^[20]^

## 1. Design and Preparation of Artificial Spidroins

**Figure 1:**
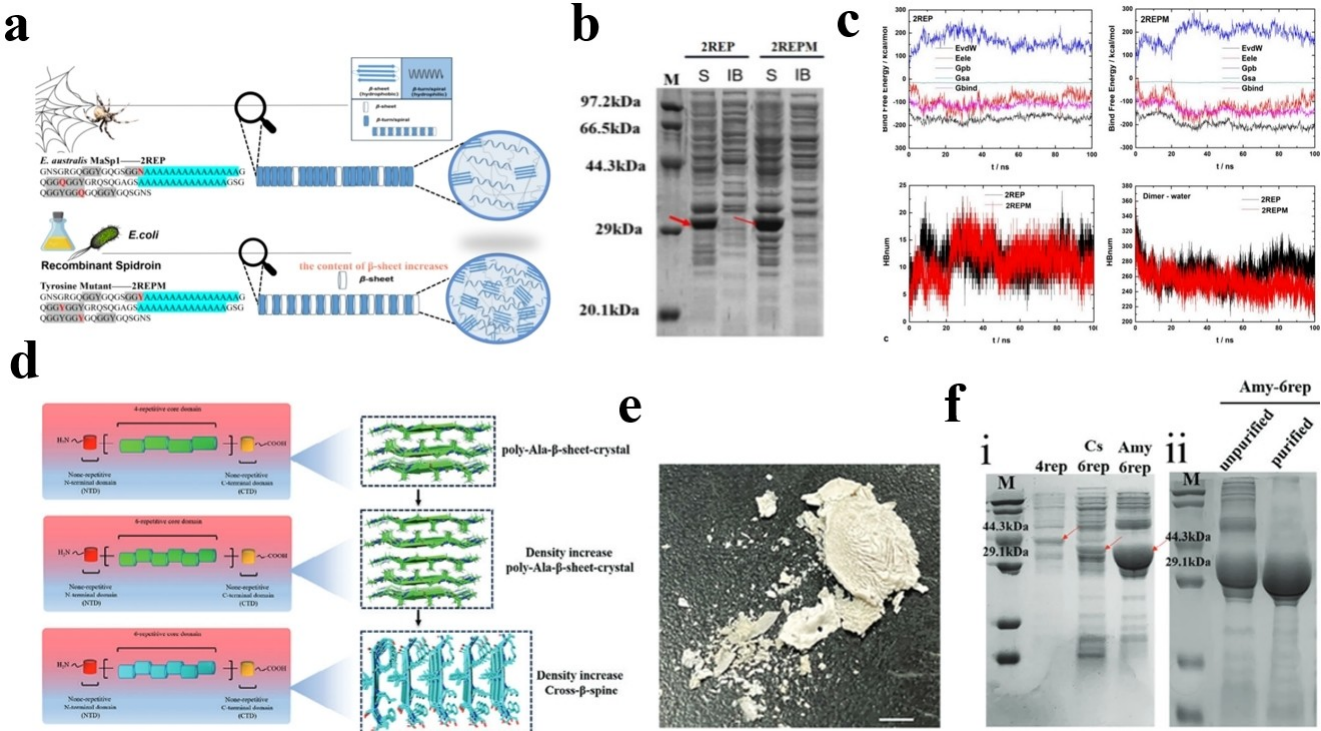
a) Schematic diagrams of the molecular structure of wild‐type spiders (2REP) and mutant spiders (2REPM) and their self‐assembled spiders. b) Expression of chimeric spidroins 2REP and 2REPM with tyrosine mutations (MW: 33.2 kDa). c) Relationship between the binding free energy of the 2REP dimer and 2REPM dimer and the change in time. The number of hydrogen bonds between 2REP/2REPM dimers and between 2REP/2REPM dimers and aqueous solvents. Reprinted with permission from Ref. [21] copyright 2024 American Chemical Society. d) To reduce the difficulty of expression and enhance the mechanical properties of the protein, the characteristic polyalanine motif was replaced by an amyloid polypeptide sequence with a cross β‐spine structure. e) Solid powder of artificial spidroin. f) SDS−PAGE analysis of artificial spidroins. Note: 4rep (primarily artificial spidroin, 40.4 kDa); Cs‐6rep (artificial spidroin with an increased number of microcrystalline regions, 39.8 kDa); Amy‐6rep (artificial spidroin with increased microcrystalline regions and amyloid polypeptide replacement (39.6 kDa)). (i) Protein purification diagram of Amy‐6rep (ii). Reprinted with permission from ref.,^[12]^ copyright 2024 Wiley‐VCH GmbH.

Cheng et al.^[21]^ The N‐terminal domain (NT) from *Euprosthenops. australis* MaSp1 and the C‐terminal domain (CT) from *A. ventricosus* MiSp, which are highly pH sensitive and soluble, and a short repetitive region (2Rep) from *Euprosthenops. australis* were selected to construct the chimeric artificial spidroin. On this basis, the tyrosine mutation sequence was disrupted. The models of 2REP and 2REPM (N154Y, Q174Y, and Q212Y) with dimeric forms were constructed via HDOCK. Figure 1c shows the relationship between the binding free energy and time change between the 2REP and 2REPM dimers. The average free binding energy of the 2REP dimer was −113.11 kcal/mol, whereas that of the 2REPM dimer was −145.09 kcal/mol. The dynamic simulation results suggest a stronger affinity between the 2REPM spidroin models than the wild‐type 2REP spidroin models. Notably, the contributions of the van der Waals force, electrostatic interaction force, polar solvation energy and nonpolar solvation energy to the binding free energy of the 2REPM dimer are −211.85, −93.66, 178.75 and −18.33 kcal/mol, respectively, which are greater than those of the 2REP dimer without tyrosine mutation (−171.95, −72.90, 146.95 and −15.21 kcal/mol). After sequence optimization, a significant self‐assembly performance of artificial spidroin (6.4 mg mL^−1^) was achieved (Figure 1b).

**Figure 2:**
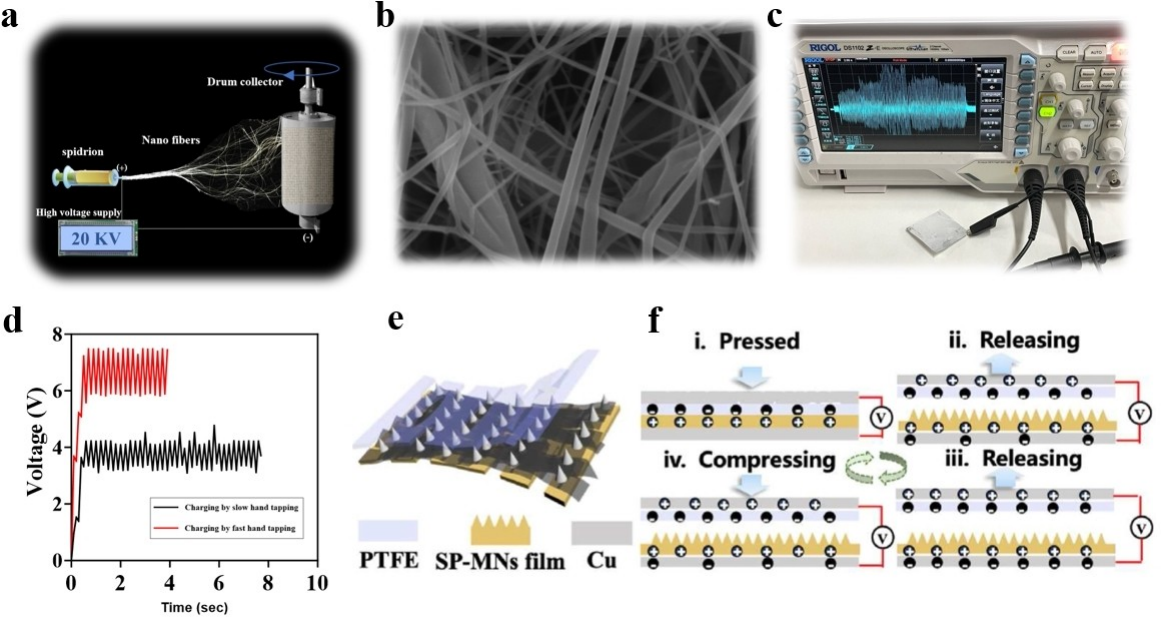
a) A nanofriction generator was prepared on the basis of artificial nanospider silk with tribopower performance via high‐pressure electrospinning technology. b) SEM image of nanospidroin fibers obtained by high‐pressure electrospinning. c) The potential generated by the friction of the nanospider fiber membrane was recorded by an oscilloscope. d) Charging of a 50 nF capacitor with nanospecidroin fibers under different rubbing frequencies. Reprinted with permission from ref.,^[12]^ copyright 2024 Wiley‐VCH GmbH. e) KSM‐TENG structure based on spider/PU‐microneedle scaffold (SP‐MNs) film, polytetrafluoroethylene (PTFE) and Cu. f) Schematic diagram of the working principle of the KSM‐TENG under the vertical contact‐separation mode. Reprinted with permission from ref.,^[26]^ copyright 2024 Elsevier.

Lin et al.^[12]^ proposed a novel and feasible method for simultaneously enhancing self‐assembly performance and obtaining high‐yield artificial spidroins. They used NT‐4RepCT (40.4 kDa, a mini chimeric spidroin) as the template. Within a finite sequence length, the number of polyalanine motifs decreases to 7; at the same time, the number of polyAla blocks increases to 6. Finally, as shown in Figure 1d, the sequential modification artificial spidroin NT‐Cs‐6rep‐CT (39.8 kDa) was obtained. The microcrystalline region of NT‐Cs‐6rep‐CT was subsequently substituted with amyloid, which is capable of forming a highly ordered cross*‐β*‐spine structure, leading to the production of the amyloid artificial spidroin Amy‐6rep. Owing to the amyloid protein sequence modification, the expression of Amy‐6rep increased by ≈200 % (Figure 1f). The protein powder, as shown in Figure 1e, can dissolve in HFIP (hexafluoroisopropanol). After electrospinning, the artificial spidroin Amy‐6rep could form nanofibers of relatively uniform diameter (Figure 2a and b).

## 2. Preparation of Artificial Spidroin Nanogenerator Wound Dressings

Artificial spider silk has outstanding mechanical properties and biocompatibility. However, current research on the functional materials of spidroins has focused primarily on enhancing the mechanical properties of spider silk fibers by stacking repeated modules from the same spider.^[22]^ There is limited research on composite microstructure design, hindering its application in more intricate and diverse scenarios and thus severely restricting the use of spidroin materials. The tensile properties of patches and dressings prepared with spidroins are guaranteed, but these materials may be too simple, which strongly limits their application. However, a layer of conductive materials can be used to form electrodes and add circuits or other microelectronic devices, such as microgenerators and sensors.[^13^, ^23^, ^24^]

Since the Teng can efficiently convert the stimulation caused by mechanical motion into electrical signals, its application in self‐powered motion sensing has attractive prospects. Li et al.^[25]^ adopted a TENG device combining SP and PEFE films with a kirigami structure, making it flexible enough to accommodate the human body. The somatosensory ability of the Teng was studied by attaching the flexible device to different locations of the body (Figure 2e and f). The KSM‐TENG patch is located on the finger, wrists and elbows. Then, electrical energy is generated through the biomechanical movement of bending at different angles; therefore, the corresponding voltage changes can be recorded and evaluated stably. In addition to monitoring the tiny dynamic movement, the patch was placed on the knee to collect the signal of bending release behavior to obtain the real‐time response at the joint.[^26^, ^27^] Cheng et al.^[17]^ integrated microchannels and MXene‐based microcircuits on SP thin films. A series of i‐SPTs with patterned microcircuits were designed and woven. It was directly connected to the fingers, wrists and elbow joints of the volunteers for motion sensing. With the gradual increase in the bending angle, the real‐time resistance also increases. Similarly, when the SP adhered to the wrist, the resistance of the SP film also tended toward good sensing ability. In response to the deformation of the wrist and elbow, the resistance of the SP film rapidly and stably changed. These results show that the SP has high sensitivity and stable motion detection ability in dynamic activities. Moderate electrical stimulation has been shown to be beneficial for wound recovery by promoting blood circulation, reducing swelling and analgesia, preventing wound infection, and effectively promoting wound healing, and the application of nanofriction generators in medical applications and wound management has often been reported. Lin et al.^[12]^ constructed artificial spidroin Amy‐6rep by charging a 50 NF capacitor through nanofriction fibers under slow and fast hand friction (Figure 2c and d). This is a more effective type of electrospun fiber for wound management. We have reviewed the abovementioned preparation methods for artificial spidroin nanogenerator‐based wound dressings, as shown in Table 1.

**Table 1:** Summary of preparation methods for the fabrication of artificial spidroin nanogenerator‐based wound dressings.

Material	Fabrication method	Form	Properties	Ref.
	Mold curing, laser cutting, crimping	Composite multilayer film, microneedle, Kirigrami structure, patch	Nanofriction generation, capillary fluidity	[17–19]
Artificial Spidroins	electrostatic spinning	Artificial nano spider silk fibers	Nanofriction generation, self‐healing property, cell adhesion	[11,21]
	Composite multilayer film, microcolumn array, electronic skin	Mold curing, laser cutting, crimping	Nanofriction generation, electric conduction	[16,20]

In summary, owing to the good biocompatibility, biodegradability, cell adhesion^[28]^ and excellent bioelectronic performance of artificial spidroins, articulus wound dressings based on spidroins have unique advantages in medical applications and wound management. The main types of medical materials prepared by artificial spidroins include films, array microneedles, nanofibers prepared by electrospinning, wet spinning and artificial spinning. Owing to the characteristics of artificial spidroins, these materials have significant advantages over traditional materials in wound management and treatment, such as excellent mechanical properties, wound permeability and hemostasis. This means more diverse applications of wound management materials. In conclusion, wound healing materials prepared from artificial spidroins have great potential.

## Conflict of Interests

The authors declare that they have no conflicts of interest.

## Biographical Information

*Xiaoming Ma is currently working in Taizhou People′s Hospital, he is also pursuing master‘s degree in Yanghozu University and Nanjing Tech University. His reserch interests are wound healing*.